# Intrathecal Fibrinolysis for Aneurysmal Subarachnoid Hemorrhage: Evidence From Randomized Controlled Trials and Cohort Studies

**DOI:** 10.3389/fneur.2019.00885

**Published:** 2019-08-19

**Authors:** Xiaocheng Lu, Chengyuan Ji, Jiang Wu, Wanchun You, Wei Wang, Zhong Wang, Gang Chen

**Affiliations:** Department of Neurosurgery & Brain and Nerve Research Laboratory, The First Affiliated Hospital of Soochow University, Suzhou, China

**Keywords:** aneurysmal subarachnoid hemorrhage, intracisternal fibrinolysis, intraventricular fibrinolysis, delayed ischemic neurological deficit, meta-analysis

## Abstract

**Background:** The role of intrathecal fibrinolysis for the treatment of patients with aneurysmal subarachnoid hemorrhage (aSAH) has been widely investigated; however, the results have been contradictory. In our study, we conducted a meta-analysis to evaluate the safety and efficacy of intrathecal (intracisternal or intraventricular) fibrinolysis for aSAH.

**Methods:** PubMed, Web of Science, Embase, Medline, and the Cochrane library databases were searched up to February 1, 2019. The outcomes analyzed were neurologic recovery, delayed ischemic neurologic deficit (DIND), mortality, and the incidence of chronic hydrocephalus and hemorrhage.

**Results:** A total of 21 studies comprising 1,373 patients were analyzed, including nine randomized controlled trials (RCTs) and 12 non-RCTs. The results showed that intracisternal fibrinolysis significantly decreased poor neurologic outcomes (RR = 0.62, 95% CI = 0.50–0.76, *P* < 0.001) and reduced the incidence of DIND (RR = 0.52, 95% CI = 0.41–0.65, P <0.001), chronic hydrocephalus (RR = 0.59, 95% CI = 0.42–0.82, *P* = 0.002) and mortality (RR = 0.58, 95% CI = 0.37, 0.93, *P* = 0.02). There was no significant difference in the occurrence of hemorrhage. Moreover, the results of the Egger test and Begg's funnel plot showed no evidence of publication bias.

**Conclusions:** Current evidence suggests that intracisternal fibrinolysis has beneficial effects on the clinical outcomes of patients with aSAH. However, further well-designed randomized trials are needed to confirm the efficacy and safety of intracisternal fibrinolysis for the treatment of aSAH.

## Introduction

Despite significant progress in the reduction of mortality, survivors of aneurysmal subarachnoid hemorrhage (aSAH) still experience long-term cognitive and functional limitations (1, 2). Cerebral vasospasm and delayed ischemic neurologic deficit (DIND) are the two major contributors to secondary morbidity and mortality following severe aSAH (3–6). Moreover, hydrocephalus, another common cause of neurologic deterioration, is associated with worse outcomes (7). Previous studies indicate that the volume of SAH and intraventricular hemorrhage (IVH), as well as the clearance rate of clots, were independent predictors of poor neurologic outcomes (8, 9). In this respect, intrathecal fibrinolysis using tissue plasminogen activator (tPA) or urokinase (UK) to reduce subarachnoid or intraventricular blood was reported to be beneficial, reducing poor neurologic outcomes in patients with aSAH (10–13). However, the results of published randomized and observational trials failed to consistently substantiate the efficacy of thrombolytic agents in preventing DIND and poor clinical outcomes (14–16). A recent meta-analysis investigated the effect of intrathecal fibrinolysis in patients with aSAH (17), however, several recent RCTs were not included (15–18). Moreover, one study described as “randomized” in a previous meta-analysis was not classified as a RCT in our meta-analysis because the authors did not offer details regarding their methodology (19). Because of these shortcomings, we performed a systematic review and meta-analysis to assess the effect of intrathecal fibrinolysis on neurologic outcomes, DIND, and mortality as well as the resulting complications, including hemorrhage and chronic hydrocephalus.

## Methods

### Search Strategy

This systematic review and meta-analysis were conducted in accordance with the guidelines of PRISMA (Preferred Reporting Items for Systematic Reviews and Meta-Analyses) (20). Our comprehensive electronic search of PubMed, Web of Science, Embase, Medline, and the Cochrane library databases was performed using the following search terms: thrombolytic therapy, tPA, fibrinolytic agents, urokinase, SAH, subarachnoid hemorrhage, DIND, delayed cerebral ischemia, clinical vasospasm, and cerebral aneurysm (the last search update was made on February 1, 2019). In addition, the references of all retrieved articles were checked for possible additional studies.

### Inclusion and Exclusion Criteria

The inclusion criteria were as follows: (1) publications comparing intrathecal (intracisternal or intraventricular) thrombolytic therapy with a control treatment in patients with aSAH and (2) papers assessing outcomes in terms of the development of DIND, the Glasgow Outcome Scale (GOS) score, modified Rankin Scale (mRS) score, mortality, chronic hydrocephalus, hemorrhage, and/or a rebleeding complication.

Articles were excluded if (1) a control group was unavailable or (2) no information regarding outcomes was provided. We also excluded case reports, review articles, articles comprised of abstracts only, meta-analyses, and duplicate reports.

### Data Extraction

For each eligible study, the following information was abstracted by two independent authors using a standardized data extraction form comprising study design, sample size, patient eligibility criteria, duration of follow-up, treatment group criteria, patient characteristics, sex and age of patients, severity of hemorrhage (indicated by Fisher grade), neurologic grade (indicated by World Federation of Neurosurgical Societies [WFNS]) criteria or Glasgow Coma Scale [GCS] score), interventions (type, timing, dose, and method of delivery of the fibrinolytic agents and concomitant therapies), and outcomes (development of DIND, GCS score, mRS score, mortality, chronic hydrocephalus, hemorrhage and/or rebleeding complications). Disagreements were resolved by discussion with a third author.

### Quality Assessment

The quality of all eligible studies was assessed by two independent authors. For RCTs, the Cochrane Collaboration's tool was used to assess risk of bias according to the following domains: selection bias (random sequence generation and allocation concealment), attrition bias (incomplete outcomes data), performance and detection bias (blinding of participants, personnel, and outcome assessment), reporting bias (selective reporting), and other sources of bias (21). In addition, the Newcastle–Ottawa Scale (NOS) was used to assess the risk of bias in the non-RCTs (22).

### Data Analysis and Statistical Methods

The following outcomes analyses were included: association of intrathecal (intracisternal or intraventricular) fibrinolysis following aSAH with (1) neurologic recovery (defined as “poor” if patients with Glasgow Outcome Scale (GOS) score between 1 and 3 and/or a mRS score between 4 and 6); (2) DIND, which was defined according to the criteria of individual papers; (3) mortality; (4) chronic hydrocephalus, defined as the need for a permanent shunt; and (5) hemorrhage and/or rebleeding complications.

Between-study heterogeneity was assessed by the χ^2^-based Q test and *I*^2^ test. Heterogeneity was considered significant when the *P*-value was less than 0.1 or *I*^2^ was >50%, then pooled risk estimates were calculated using the random-effects model (DerSimonian-Laird); otherwise, a fixed-effects model (Mantel-Haenszel) was used (23, 24).

Assessment of publication bias was performed using a graphic evaluation of funnel plots and the Egger regression test. A *P*-value of less than 0.05 from the Egger test was considered statistically significant (25). All statistical analyses were performed using Review Manager (RevMan) (version 5.2, The Nordic Cochrane Centre, The Cochrane Collaboration, Copenhagen, 2012) and Stata (version 12 software, StataCorp LP, College Station, TX, USA).

## Results

### Characteristics of Included Studies

**Figure 1:**
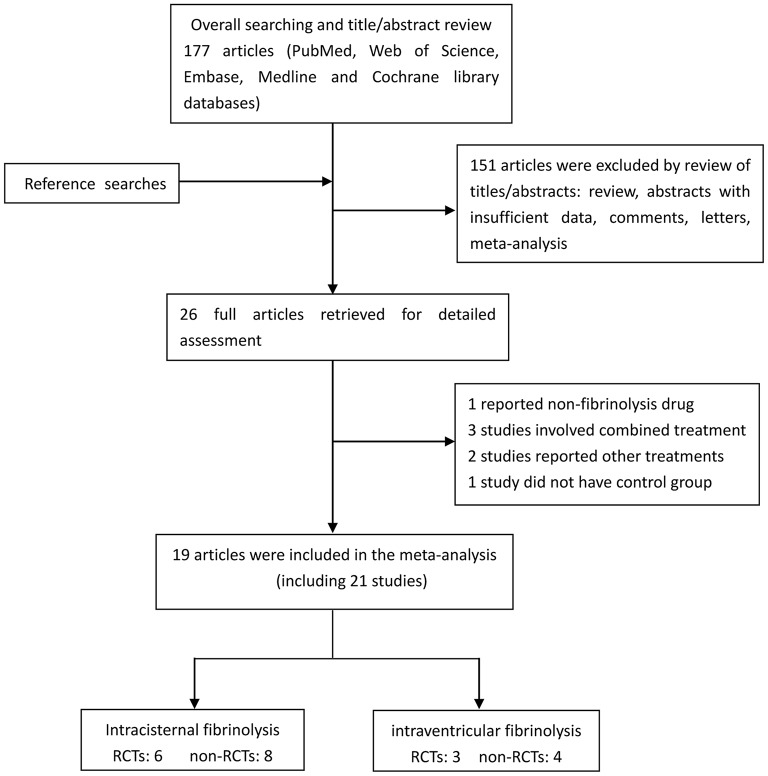
Flow diagram showing the selection of eligible studies.

A flow diagram detailing the study selection is shown in Figure 1. Briefly, the literature search produced 177 citations, of which 151 were excluded by review of the abstracts. Thereafter, full texts of the remaining 26 articles were analyzed and reviewed in detail. Finally, a total of 19 studies comprising 1,373 aSAH patients met our inclusion criteria (10–16, 18, 19, 26–35). Among these studies, one reported data on intermittent and continuous fibrinolysis, and another allocated patients to therapy with tPA or UK; that is, we treated them independently (11, 28). In total, nine RCTs and 12 non-RCTs were analyzed. The main characteristics of the studies included in this meta-analysis are summarized in Table 1.

**Table 1:** Characteristics of studies included in the meta-analysis.

**References**	**Country**	**Design**	**No (male%)**	**Fisher 3–4 (%)**	**IVH (%)**	**Clipping(%)**	**Delivery/Thrombolytic**	**Primary outcome**
Kramer et al. (15)	Canada	RCTs	12 (25.0)	100.0	100.0	0.0	IV (intraventricular via EVD), tPA: 2 mg every 12 h × 5 doses	Rate of intracranial blood clearance, feasibility, safety
Etminan et al. (16)	Germany	RCTs	60 (36.7)	96.7	55.5	60.0	IV (intraventricular via EVD), rt-PA: 5 mg every 12 h × 2 days	Functional outcome after 3 months
Litrico et al. (18)	France	RCTs	19 (60.0)	nr	100.0	5.3	IV (intraventricular via EVD), rt-PA: 3 mg every 12 h × 6 days	Mortality rate within 30 days
Yamamoto et al. (11)	Japan	RCTs	40 (32.5)	85.0	nr	100.0	IC (basal cisterns via the cisternal drainage tube), TK: 1920 IU/h × 2 days	nr
Yamamoto et al. (11)	Japan	RCTs	40 (35.0)	87.5	nr	100.0	IC (basal cisterns via the cisternal drainage tube), TK: 160,000 IU every 8 h × 2 days	nr
Hänggi et al. (14)	Germany	RCTs	20 (45.0)	100.0	nr	60.0	IC (lumbar cistern), UK: 120,000 IU/d × 2 days	Appearance of DIND
Li et al. (35)	China	RCTs	134 (72.4)	nr	nr	nr	IC (lumbar cistern), UK: 6000–8000 IU/d*3–12 days	nr
Hamada et al. (10)	Japan	RCTs	110 (34.5)	87.3	nr	0.0	IC (cisterna magna via microcatheter), UK: 60,000 IU every 12 h × 2 doses	Symptomatic cerebral vasospasm
Findlay et al. (34)	Canada	RCTs	100 (37.0)	nr	22.0	100.0	IC (basal cisterns during operation), rt-PA: 10 mg once	Angiographic vasospasm
Gerner et al. (31)	Germany	R	88 (27.3)	8.0	88.6	0.0	IV (intraventricular via EVD), rt-PA: 1 mg/8 h until clot-clearance of 3rd and 4th ventricles	nr
Ramakrishna et al. (32)	USA	R	76 (19.7)	nr	nr	50.7	IV (intraventricular), tPA: 5 mg daily × 7 doses	nr
Varelas et al. (33)	USA	P	20 (60.0)	nr	100.0	45.0	IV (intraventricular via intraventricular catheter), tPA: 3.5 ± 2.5 mg	Resolution of blood in the third and fourth ventricles
Findlay et al. (30)	Canada	P	30 (50)	nr	100.0	100.0	IV (intraventricular via EVD), rt-PA: A initial dose of 4 mg and 2–4 mg daily	Resolution of intraventricular blood clot
Yamada et al. (29)	Japan	R	69 (40.6)	100.0	nr	75.4	IC (the chiasmatic or prepontine cisterns via cisternal drainage), UK: 10000 IU × 3–6 doses in 2–3 days	Functional outcome after 3 months
Gorski et al. (13)	Poland	R	45 (nr)	nr	nr	nr	IC (basal cisterns during operation), t-PA: 10 mg	nr
Moriyama et al. (27)	Japan	R	44 (50.0)	nr	nr	100.0	IC (chiasmatic or prepontine cistern via cisternal drainage), UK: 60000 IU × 2–4 doses	Functional outcome after 6 months
Seifert et al. (12)	Germany	P	120 (nr)	100.0	17.5	100.0	IC (basal cisterns during operation), rt-PA: 10 mg	Functional outcome after 3 months
Mizoi et al. (26)	Japan	P	105 (40.0)	100.0	nr	100.0	IC (carotid and sylvian cisterns via cisternal drainage), t-PA: 2 mg/ until all of the cisterns exhibited low density on CT scans	Functional outcome after 1 months
Usui et al. (28)	Japan	R	51 (41.2)	70.6	19.6	100.0	IC (basal or prepontine cisterns via cisternal drainage), t-PA: 0.042 to 1 mg/ 6–8 h × 5 days	Functional outcome after 3 months
Usui et al. (28)	Japan	R	89 (39.3)	76.4	10.1	100.0	IC (basal or prepontine cisterns via cisternal drainage), UK: 60000 IU/day × 5–7 days	Functional outcome after 3 months
Kanamura et al. (19)	Japan	P	101 (nr)	96.0	nr	100.0	IC (basal cisterns via microcatheter), UK: 60000 IU/d	Functional outcome after 3 months

*UK, urokinase; TK, Tisokinase; R, retrospective; P, prospective; RCTs, randomized controlled trials; Data are number (%), mean (SD) or median (range); IC, intracisternal fibrinolysis; IV, intraventricular fibrinolysis; n.r., not reported*.

### Risk of Bias

**Figure 2:**
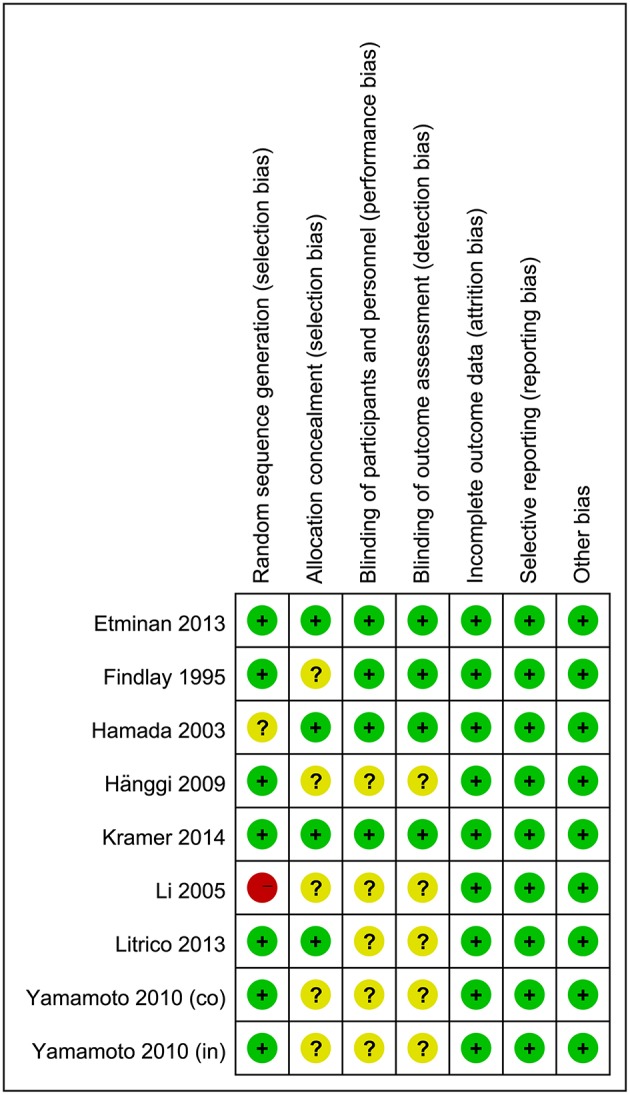
Risk-of-bias assessment for randomized controlled trials. A plus sign (+) indicates a low risk of bias, a minus sign (–) indicates a high risk of bias, and a question mark (?) indicates an unclear risk of bias.

The results of quality assessments for RCTs are summarized in Figure 2. Briefly, randomization methods were not proper in one study and not described in another. The allocation concealments were not adequate in five studies. Moreover, five studies did not describe the blinding of outcomes assessment and participants. For cohort studies, quality assessments were made by using NOS, and the results showed that five studies had a low risk of bias (8 out of 9 points) and seven studies had a moderate risk of bias (6–7 out of 9 points) (Supplementary Tables 1, 2).

### Neurologic Recovery and DIND

**Figure 3:**
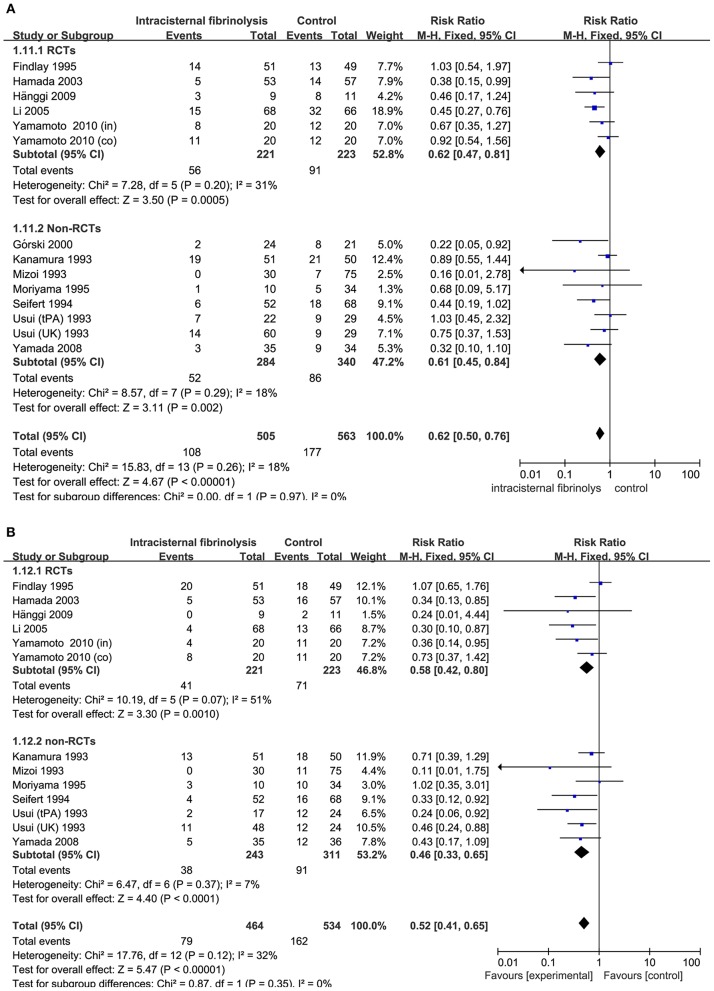
Meta-analysis of associations between intracisternal fibrinolysis and the risk of poor neurologic recovery **(A)** or the incidence of DIND **(B)** in patients with aSAH. aSAH, aneurysmal subarachnoid hemorrhage; CI, confidence interval; DIND, delayed ischemic neurologic deficit; M-H, Mantel-Haenszel method; RCTs, randomized controlled trials.

**Figure 4:**
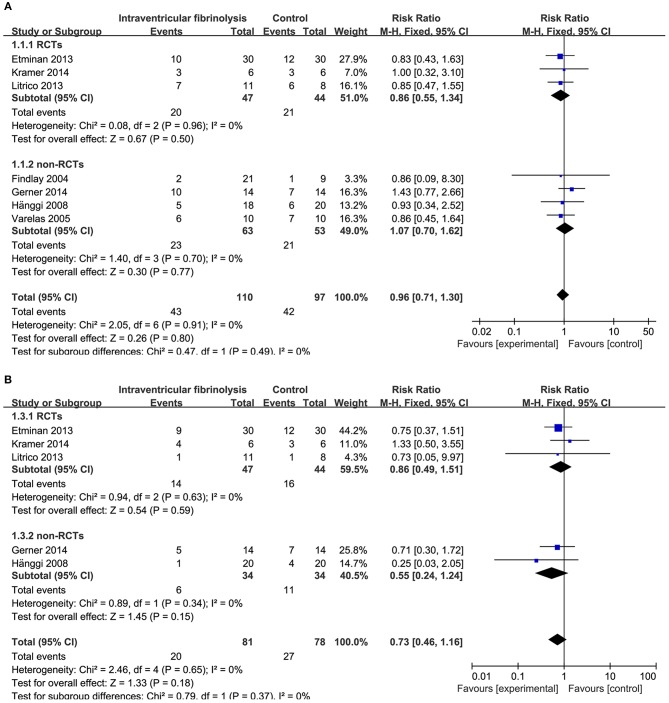
Meta-analysis of associations between intraventricular fibrinolysis and the risk of poor neurologic recovery **(A)** or the risk of DIND **(B)** in aSAH patients. aSAH, aneurysmal subarachnoid hemorrhage; CI, confidence interval; DIND, delayed ischemic neurologic deficit; M–H, Mantel–Haenszel method; RCTs, randomized controlled trials.

Combining the results of all 14 studies (6 RCTs and 8 non-RCTs), treatment with intracisternal fibrinolysis significantly reduced the proportion of patients with poor neurologic recovery (RR = 0.62, 95% CI = 0.50–0.76, *P* < 0.001 for overall analysis; RR = 0.62, 95% CI = 0.47–0.81, *P* < 0.001 for the RCT group; RR = 0.61, 95% CI = 0.45–0.84, *P* < 0.001 for the non-RCT group; Figure 3A). The overall incidence of DIND was 17.0% in the intracisternal fibrinolysis group as compared with 30.3% in the control group. The pooled relative risk of DIND was 0.52 (95% CI = 0.41–0.65) for the overall analysis (in the subgroup analysis, RR = 0.58, 95% CI = 0.42–0.80, *P* < 0.001 for the RCT group; RR = 0.46, 95% CI = 0.33–0.65, *P* < 0.001 for the non-RCT group; Figure 3B). For intraventricular fibrinolysis, no significant improvements in poor neurologic outcome and DIND were observed in either RCTs (RR = 0.86, 95% CI = 0.55–1.34, *P* = 0.50 and RR = 0.86, 95% CI = 0.49–1.51, *P* = 0.59, respectively) or non-RCTs (RR = 1.07, 95% CI = 0.70–1.62, *P* = 0.77 and RR = 0.55, 95% CI = 0.24–1.24, *P* = 0.15, respectively; Figures 4A,B).

**Figure 5:**
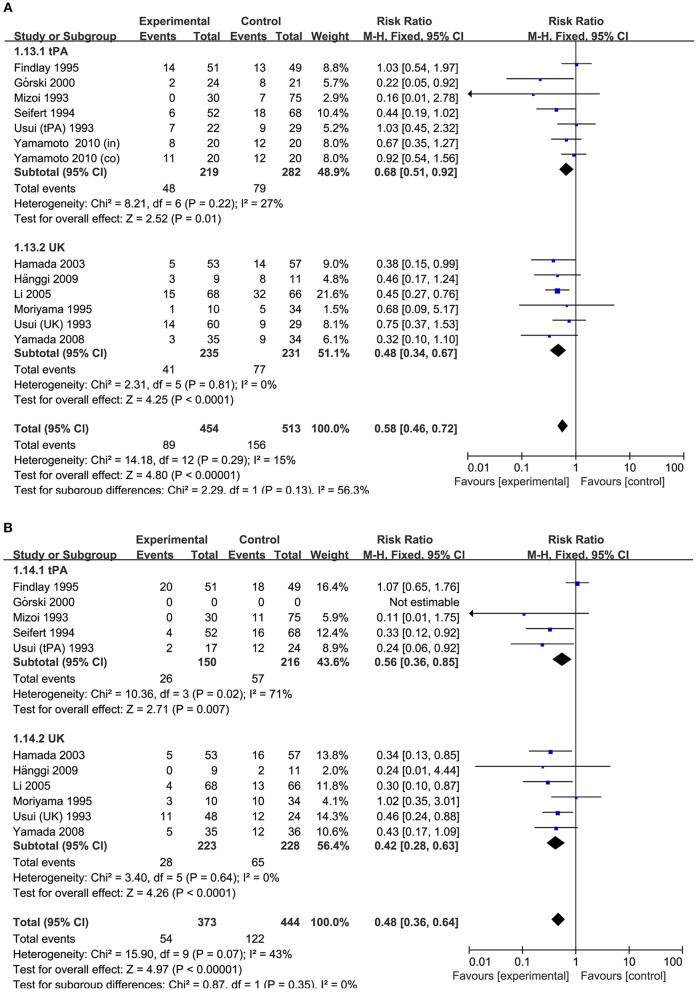
Meta-analysis of associations between intracisternal fibrinolysis and the risk of poor neurologic recovery **(A)** or the incidence of DIND **(B)** in patients with aSAH (stratified by types of thrombolytic agents). aSAH, aneurysmal subarachnoid hemorrhage; CI, confidence interval; M–H, Mantel–Haenszel method; UK, urokinase; tPA, tissue plasminogen activator.

In the subgroup analysis stratified by types of thrombolytic agents, the results showed that either intracisternal tPA or UK infusion significantly decreased the risk of pool neurologic outcomes (RR = 0.68, 95% CI = 0.51–0.92, *P* = 0.01 and RR = 0.48, 95%CI = 0.34–0.67, *P* < 0.0001, respectively; Figure 5A) and DIND (RR = 0.56, 95%CI = 0.36–0.85, *P* = 0.005 and RR = 0.42, 95% CI = 0.28–0.63, *P* < 0.001, respectively; Figure 5B). Moreover, the subgroup analysis by the methods of aneurysm treatment (clipping or coiling) showed intracisternal fibrinolysis significantly reduced the proportion of patients with poor neurologic recovery in the clipping subgroup (RR = 0.77, 95% CI = 0.61–0.98, *P* = 0.04). However, there was only one study reporting the patients with aSAH treated with coiling (RR = 0.38, 95% CI = 0.15–0.99, *P* = 0.05) (10). For intraventricular fibrinolysis, there were insufficient data to perform a subgroup analysis by types of thrombolytic agents and the methods of aneurysm treatment.

### Mortality and Chronic Hydrocephalus

For intracisternal fibrinolysis, a meta-analysis of 11 studies (including five RCTs and six non-RCTs) showed that intracisternal fibrinolysis significantly decreased mortality among patients with severe SAH (RR = 0.58, 95% CI = 0.37, 0.93, *P* = 0.02) compared with patients receiving conventional treatment. However, the results of a pooled analysis did not show any difference in mortality between the intraventricular fibrinolysis group and the control group (RR = 0.76, 95% CI = 0.46, 1.26, *P* = 0.28).

**Figure 6:**
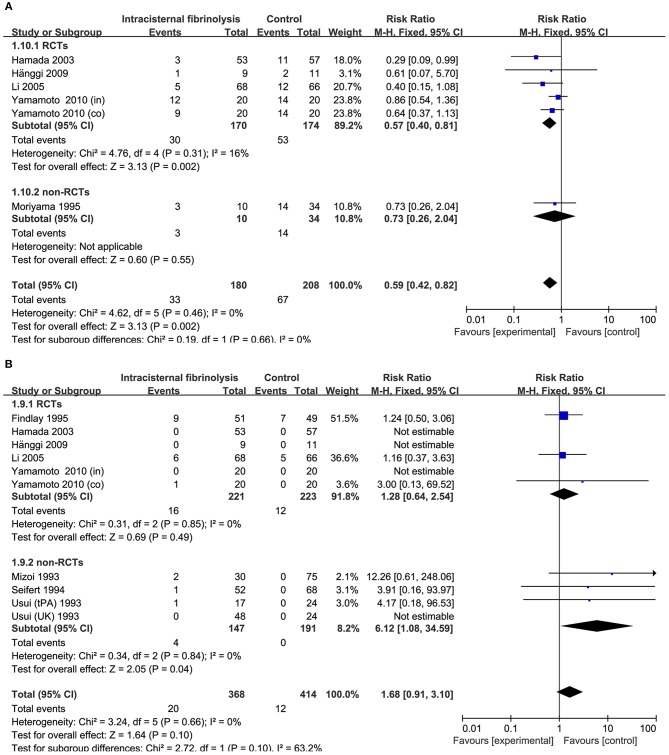
Meta-analysis of associations between intracisternal fibrinolysis and the risk of chronic hydrocephalus **(A)** or the risk of hemorrhagic complications **(B)** in patients with aSAH. aSAH, aneurysmal subarachnoid hemorrhage; CI, confidence interval; M–H, Mantel–Haenszel method; RCTs, randomized controlled trials.

**Figure 7:**
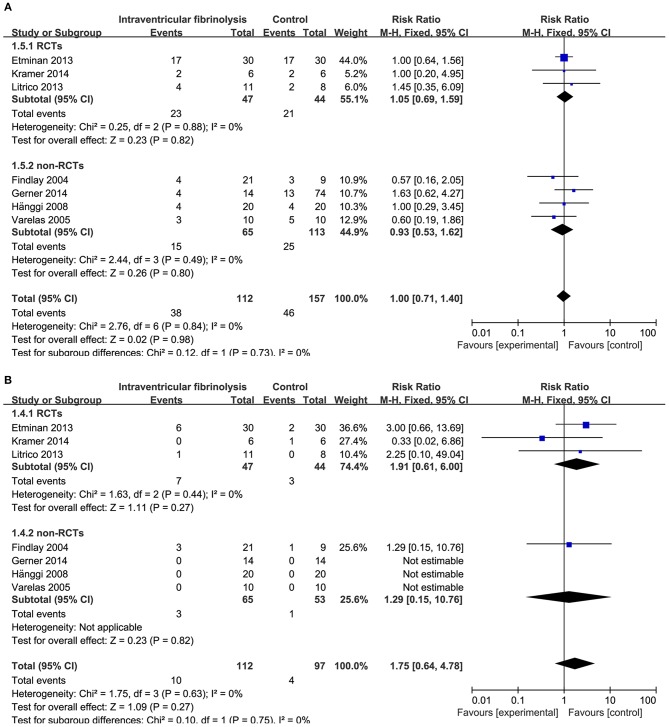
Meta-analysis of associations between intraventricular fibrinolysis and the risk of chronic hydrocephalus **(A)** or the risk of hemorrhagic complications **(B)** in patients with aSAH. aSAH, aneurysmal subarachnoid hemorrhage; CI, confidence interval; M–H, Mantel–Haenszel method; RCTs, randomized controlled trials.

The risk of chronic hydrocephalus, defined as the need for a permanent shunt, was significantly decreased in patients treated with intracisternal fibrinolysis (RR = 0.59, 95% CI = 0.42–0.82, *P* = 0.002; Figure 6A). However, for intraventricular fibrinolysis-treated patients, no significant association was observed between fibrinolytic therapy and chronic hydrocephalus in overall estimation (RR = 1.00, 95% CI = 0.71–1.40, *P* = 0.98), as well as in RCTs (RR = 1.05, 95% CI = 0.69–1.59, *P* = 0.82) or non-RCT subgroups (RR = 0.93, 95% CI = 0.53–1.62, *P* = 0.80; Figure 7A).

### Hemorrhagic Complications

Hemorrhagic complications were described in 10 studies for intracisternal fibrinolysis. The results of a pooled analysis demonstrated that hemorrhagic complications were not increased overall (RR = 1.68, 95% CI = 0.91–3.10, *P* = 0.10) or in RCT subgroups (RR = 1.28, 95% CI = 0.64–2.54, *P* = 0.49), whereas which was significantly increased in non-RCTs subgroups (RR = 6.12, 95% CI = 1.08-34.59, *P* = 0.04; Figure 6B). For intraventricular fibrinolysis, seven studies reported the incidence of hemorrhagic complications. After combining the results of these studies, a difference in the rate of hemorrhage between intraventricular fibrinolysis and the control groups was not shown (RR = 1.75, 95% CI = 0.64–4.78, *P* = 0.27; Figure 7B).

### Test of Heterogeneity and Publication Bias

Between-study heterogeneity was assessed by the χ^2^-based Q and *I*^2^ tests. The results revealed non-significant heterogeneity in all analyses (*P* > 0.2 for all analyses). For publication bias, the shapes of the funnel plots did not show evidence of obvious asymmetry in any of the studies included in the pooled analysis (Supplementary Figure 1). In addition, we performed an Egger test to provide statistical evidence of funnel plot symmetry, which supported the results of the funnel plots.

## Discussion

Although the subarachnoid clot volume and clot clearance rate were important risk factors for the development of DIND and hydrocephalus following a ruptured cerebral aneurysm, it remained uncertain whether therapies aimed to the clearance of blood improved outcomes in aSAH patients (7, 8). By combining the results of nine RCTs and 12 retrospective or prospective studies involving a total of 1,373 patients, we found that intracisternal fibrinolysis reduced the incidence of DIND, chronic hydrocephalus, and mortality and also improved functional recovery in patients with aSAH. There was no significant difference in rebleeding complications between the fibrinolysis and control groups.

DIND is a distinctive syndrome of cerebral ischemia after SAH; it is a major cause of mortality and disability and is difficult to treat (36, 37). One reason for this difficulty is that the exact pathophysiology of DIND is unclear. Recent research has shown that vasospasm is not the only cause of DIND (38). Regional hypoperfusion often occurs in territories without angiographic vasospasm, and other factors—such as early brain injury, intravascular inflammation, and microthrombosis—have been reported to cause DIND (39, 40). Previously published studies reported a rate of 33% to 38% for DIND after SAH (41). In our study, we found that intracisternal fibrinolysis significantly decreased the occurrence of DIND (by 17.0 and 30.3% in the intracisternal fibrinolysis and placebo-treated patients, respectively), whereas no significant difference was observed in the intraventricular fibrinolysis group.

Chronic hydrocephalus is a well-known post-aSAH complication (42). According to recent studies, the rate of chronic hydrocephalus following aSAH requiring shunt placement has ranged from 17.2 to 31.2% (43, 44). Moreover, patients who developed hydrocephalus following aSAH had a worse prognosis than those who did not (45). We evaluated the association between intrathecal fibrinolysis and chronic hydrocephalus as reported in 13 studies (seven focusing on intraventricular fibrinolysis and six focusing on intracisternal fibrinolysis). The results showed a significant decrease in the risk of chronic hydrocephalus in aSAH patients treated with intracisternal fibrinolysis. One theory explaining the pathogenesis of hydrocephalus after aSAH is that subarachnoid blood interferes with the circulation of cerebrospinal fluid (CSF) at the basal cisterns, the foramen of Monro, or throughout the subarachnoid space (46, 47). Intracisternal fibrinolysis reduced the hematoma in the basal cisterns at 48 h after aSAH, which might have lowered the risk of hydrocephalus (10).

Another consideration is the safety of intrathecal fibrinolysis. We therefore investigated whether fibrinolysis led to a rebleeding complication after aSAH. In the overall analysis, the pooled results showed a non-significant impact of intrathecal fibrinolysis on rebleeding complications (intracisternal fibrinolysis group vs. control group: 5.4 vs. 2.9%), whereas in the non-RCTs subgroup of intracisternal fibrinolysis, a significantly increased risk for rebleeding was observed (intracisternal fibrinolysis non-RCT group vs. control non-RCT group: 2.7 vs. 0.0%). We also found that the ratio of patients with epidural hemorrhage was 50% (two of four rebleeding patients) in the fibrinolysis group in the non-RCTs studies. It is possible that intrathecally injected tPA leaks extradurally and leads to epidural hematoma formation, which might have occurred if the dural closure was not completely watertight (12, 26, 28).

To date, the association between the type of fibrinolytic agent and outcome remains incompletely understood. Most of the thrombolytic agents used in these studies were tPA, rt-PA, and UK. Only one study allocated patients to therapy with tPA or UK; it showed that there were non-significant differences in the effect of fibrinolysis on poor neurologic outcomes or DINDs between patients treated either agent (28). We performed subgroup analyses stratified by thrombolytic agents, which showed that the rate of patients with poor neurologic outcomes or DINDs was significantly decreased in the tPA and UK groups as compared with the control groups. This result was consistent with a randomized primate study, indicating that both drugs (t-PA and UK) had similar effects on the elimination of subarachnoid clot (48).

Several potential limitations to this meta-analysis should be considered. First, the methodology of different studies varied, including the type and dosage of thrombolytic agents, whereas there were insufficient data to perform a subgroup analysis. Second, this meta-analysis included only published studies, which might introduce publication bias, although the Egger tests and funnel plot indicated no bias. Third, a recent study showed that intraventricular tPA administration might produce a transient local inflammatory response (49). However, intracranial infections were not evaluated in this meta-analysis owing to insufficient data. Finally, a language bias might be possible because only studies published in English and Chinese were included in this study.

## Conclusion

Despite these limitations, the present meta-analysis provides evidence that intracisternal fibrinolysis was effective in improving aSAH patients' functional recovery, as well as in lowering the risk of DIND, chronic hydrocephalus, and mortality. A recent prospective RCT including 440 patients is running, which aimed to investigate the effect of intraventricular fibrinolysis for aSAH (50). Moreover, further multicenter RCTs are needed to better evaluate the safety and efficacy of intrathecal fibrinolysis, including the type and dosage of thrombolytic agents and the incidence of intracranial infective complications.

## Author Contributions

ZW and GC contributed to study design, data extraction, data analysis, and manuscript drafting. XL and CJ were responsible for data extraction and data analysis. WY and JW involved in the literature search. WW was responsible for the data extraction and manuscript drafting.

### Conflict of Interest Statement

The authors declare that the research was conducted in the absence of any commercial or financial relationships that could be construed as a potential conflict of interest.
