# Supplementary material for: Intrathecal Fibrinolysis for Aneurysmal Subarachnoid Hemorrhage: Evidence From Randomized Controlled Trials and Cohort Studies
Source: Front Neurol. 2019 Aug 19;10:885. doi: 10.3389/fneur.2019.00885 (PMC6709660; doi:10.3389/fneur.2019.00885)
Supplement: Supplementary file 1 [file Data_Sheet_1.PDF]

Intrathecal Fibrinolysis for Severe Aneurysmal Subarachnoid Hemorrhage: Evidence from Randomized Controlled Trials and Cohort Studies

Xiaocheng Lu[#], Chengyuan Ji[#], Jiang Wu, Wanchun You, Wei Wang, Zhong Wang*, Gang Chen*

Department of Neurosurgery & Brain and Nerve Research Laboratory, The First Affiliated Hospital of Soochow University, Suzhou, China

[#] These authors contribute equally to this work.

* Correspondence to: Gang Chen, email: nju_nuerosurgery@163.com, or Zhong Wang, email: dr_zhongwang@126.com , Department of Neurosurgery & Brain and Nerve Research Laboratory, The First Affiliated Hospital of Soochow University, No. 188 Shizi Street, Suzhou, 215006, China (Tel: 86-512-67780967, Fax: 86-512-65238350)

Supplementary Table 1. Modified Newcastle-Ottawa Quality Assessment Scale (cohort studies)

**Assessment of quality of a cohort study – Newcastle Ottawa Scale**	
**Selection** (tick one box in each section)	Stars
1. Representativeness of the intervention cohort<br>a) Truly representative of the aSAH population<br>b) Somewhat representative of the aSAH population<br>c) Selected group of patients<br>d) No description of the derivation of the cohort	 *<br>*
2. Selection of the non intervention cohort<br>a) Drawn from the same community as the intervention cohort<br>b) Drawn from a different source<br>c) No description of the derivation of the non intervention cohort	 *
3. Ascertainment of intervention<br>a) Secure record (e.g. health care record)<br>b) Structured interview<br>c) Written self report<br>d) Other / no description	 *<br>*
4. Demonstration that outcome of interest was not present at start of study<br>a) Yes<br>b) No	 *
**Comparability** (tick one or all boxes, as appropriate)	
1. Comparability of cohorts on the basis of the design or analysis<br>**a)** Study controls for **age**<br>**b)** Study controls for **Hunt and Hess grade or World Association of Neurological Surgeons (WFNS) or Glasgow Coma Scale score**	 *<br>*
**Outcome** (tick one box in each section)	
1. Assessment of outcome<br>a) Independent blind assessment<br>b) Record linkage<br>c) Self report<br>d) Other / no description	 *<br>*
2. Was follow up long enough for outcomes to occur<br>a) Yes, if median duration of follow-up >= 6 month<br>b) No, if median duration of follow-up < 6 months	 *
3. Adequacy of follow up of cohorts<br>a) Complete follow up: all subjects accounted for<br>b) Subjects lost to follow up unlikely to introduce bias: number lost <= 20%, or description of those lost suggesting no different from those followed<br>c) Follow up rate < 80% and no description of those lost<br>d) No statement	 *<br>*

Note: A study can be awarded a maximum of one star for each numbered item within the Selection and Outcome categories. A maximum of two stars can be given for Comparability

Supplementary Table 2. Risk of bias assessment for nonrandomized cohort studies

First author, year	Selection	Comparability	Outcome	Total score
Gerner, 2014	****	*	**	7/9
Ramakrishna, 2014	***	*	**	6/9
Varelas, 2005	***	**	**	7/9
Findlay, 2004	***	*	**	6/9
Yamada, 2008	****	**	**	8/9
Górski, 2000	***	*	**	6/9
Moriyama, 1995	***	**	***	8/9
Seifert, 1994	****	**	**	8/9
Mizoi, 1993	****	*	**	7/9
Usui (tPA), 1993	****	**	**	8/9
Usui (UK), 1993	****	**	**	8/9
Kanamura, 1993	***	*	**	6/9

Supplementary Figure 1

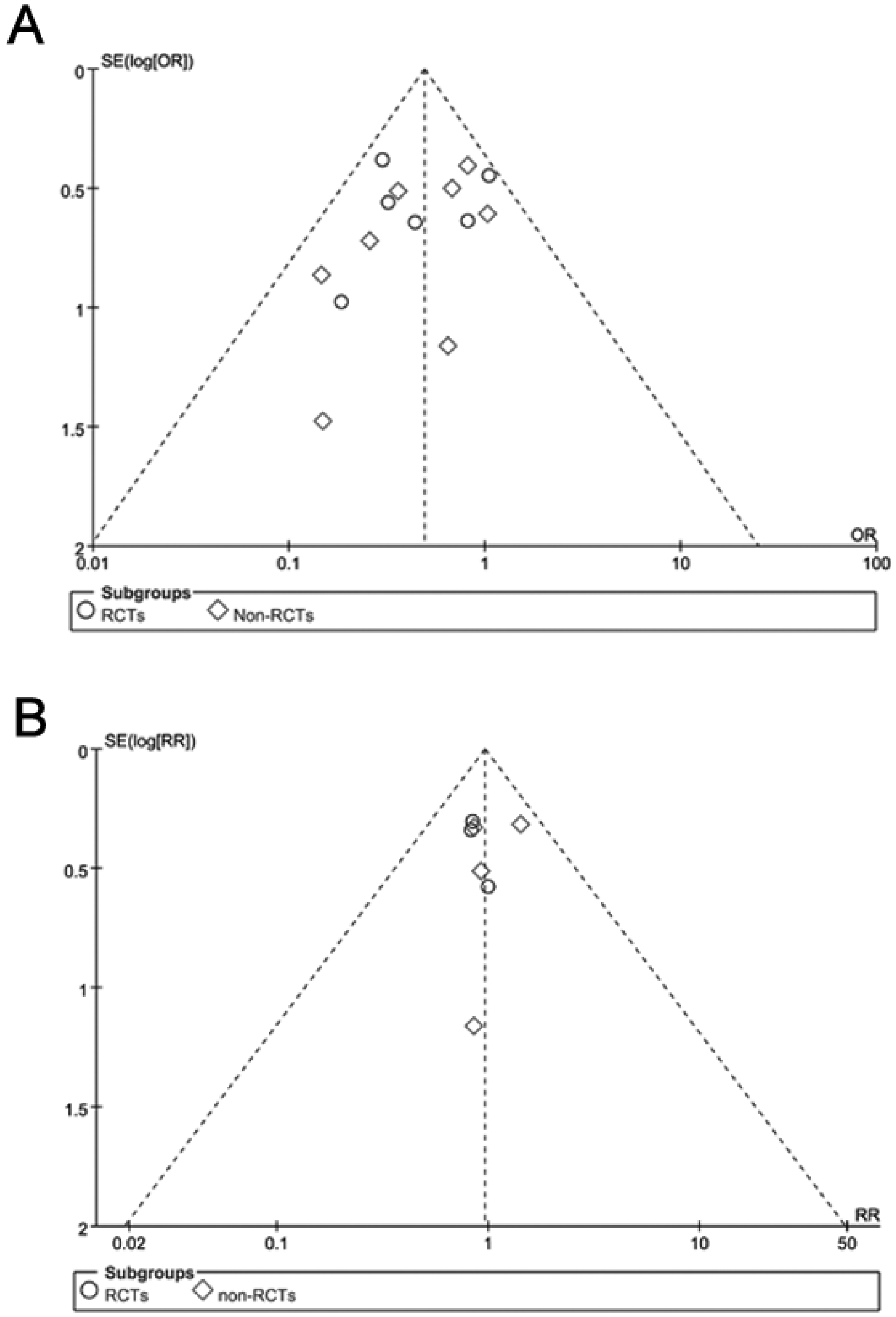

Supplementary Figure 1. Funnel plot to detect publication bias in the meta-analysis. No significant funnel asymmetry that could indicate publication bias was observed.
